# CT-based intrathrombus and perithrombus radiomics for predicting complete recanalization after endovascular thrombectomy in acute ischemic stroke

**DOI:** 10.3389/fneur.2026.1868821

**Published:** 2026-08-19

**Authors:** Qianqian Zhao, Shuaishuai Feng, Huihui Jia, Minda Li, Hao Tian, Haibin Pan, Jingxuan Jiang

**Affiliations:** 1 Institute of Diagnostic and Interventional Radiology, Shanghai Sixth People's Hospital Affiliated to Shanghai Jiao Tong University School of Medicine, Shanghai, China; 2 Department of Radiology, The Eighth People's Hospital of Jinan, Jinan, Shandong, China; 3 Department of Radiology, Huadong Hospital, Fudan University, Shanghai, China; 4 Department of Radiology, Affiliated Hospital of Nantong University, Nantong, China; 5 Department of Radiology, Shanghai Fengxian Central Hospital, Shanghai, China

**Keywords:** acute ischemic stroke, complete recanalization, endovascular thrombectomy, machine learning, perithrombus, radiomics

## Abstract

**Objective:**

To develop and validate CT-based radiomics models incorporating intrathrombus and perithrombus features for predicting complete recanalization [modified Thrombolysis in Cerebral Infarction (mTICI)2c/3] after endovascular thrombectomy (EVT) in acute ischemic stroke (AIS), and to identify the optimal machine learning classifier.

**Materials and methods:**

This retrospective study included 406 AIS patients with anterior circulation large-vessel occlusion from three centers (December 2018–April 2024). Patients were allocated to training (*n* = 178), internal testing (*n* = 77), and external validation (*n* = 151) cohorts. Complete recanalization was defined as mTICI 2c/3. A total of 428 radiomics features were extracted from non-contrast CT and CT angiography (CTA). Least absolute shrinkage and selection operator (LASSO) regression and eleven classifiers were employed.

**Results:**

The combined intrathrombus-perithrombus model with logistic regression achieved area under the curve (AUC) values of 0.93 (training), 0.88 (testing), and 0.86 (validation), outperforming single-region models. Decision curve analysis confirmed superior clinical utility. The perithrombus region contributed dominantly (10 of 15 features) to the combined model.

**Conclusion:**

The combined CT-based radiomics model effectively predicts complete recanalization, providing an objective tool for patient selection and treatment optimization.

## Introduction

Endovascular thrombectomy (EVT) is the cornerstone treatment for acute ischemic stroke (AIS) due to anterior circulation large-vessel occlusion (1). Successful recanalization is crucial for improving patient prognosis; however, recanalization rates vary significantly across studies (2–4). When EVT fails, repeated endovascular manipulations and contrast agent exposure may impair outcomes (5). Therefore, preoperative prediction of recanalization outcomes to identify optimal candidates represents a critical unmet need.

The Thrombolysis in Cerebral Infarction (TICI) scale and its extended version (eTICI) constitute the gold standard for assessing vascular recanalization after EVT (6). Patients achieving TICI grade 3 (complete recanalization) demonstrate the highest rates of favorable functional prognosis at 90 days, whereas those with eTICI grades 2c/3 (near-complete/complete recanalization) exhibit significantly better outcomes than patients with eTICI grade 2b (partial recanalization) (7, 8). Importantly, eTICI grade 2c possesses independent prognostic value and is associated with superior early neurological improvement at 24 h compared with grade 2b (8), supporting eTICI 2c/3 as the ideal angiographic target for EVT.

Recent evidence indicates that even experienced clinicians accurately predict thrombectomy outcomes in only 44% of cases (9), highlighting substantial uncertainty in clinical decision-making. Thrombus assessment is crucial for predicting EVT efficacy (10). While both CT and MRI can detect thrombi, CT is preferred in emergency settings due to its widespread availability and rapid acquisition (11). Although thrombus characteristics (composition, permeability) correlate with recanalization success (12, 13), traditional imaging relies on subjective radiologist interpretation, and no single biomarker fully satisfies clinical requirements (14).

Radiomics enables quantitative analysis of medical images by extracting high-dimensional features invisible to conventional assessment (15). In AIS, thrombus radiomics demonstrates potential for detecting thrombus composition (16, 17), predicting stroke risk (18), and classifying stroke etiology (19). Notably, our previous study established that CT-based thrombus radiomics predicts successful recanalization (mTICI > = 2b) (19). However, complete recanalization (mTICI2c/ 3) provides significantly better functional outcomes than partial recanalization (mTICI 2b) (20–22), making it a more stringent and clinically relevant endpoint. While prior research focused primarily on intrathrombus features, the perithrombus region-which reflects vessel wall pathology and thrombus-vessel interactions-remains unexplored for predicting complete recanalization. This study addresses these gaps by developing combined intrathrombus-perithrombus radiomics models specifically targeting mTICI 2c/3.

## Materials and methods

### Patients

This retrospective analysis included AIS patients from three hospitals (Affiliated Hospital of Nantong University, Shanghai Sixth People’s Hospital, Kunshan Second People’s Hospital) between December 2018 and April 2024. Inclusion criteria were: (1) AIS caused by acute anterior circulation large-vessel occlusion (internal carotid artery post-communicating segment, middle cerebral artery M1 segment); (2) thrombus-related signs visible on initial non-contrast CT (NCCT) or CT angiography (CTA); (3) EVT performed within 24 h of onset according to current guidelines (23); (4) immediate EVT following CT examination; (5) high-quality digital subtraction angiography (DSA) available for recanalization assessment; and (6) complete demographic and clinical data. Exclusion criteria were insufficient image quality precluding adequate visualization and incomplete clinical data.

Clinical variables included age, sex, medical history (hypertension, diabetes, hyperlipidemia, atrial fibrillation, and coronary artery disease), thrombus location, National Institutes of Health Stroke Scale (NIHSS) score at admission, and onset-to-puncture time (OPT). Complete recanalization was defined as mTICI score >2b (grades 2c/3) (6). A neurointerventional radiologist with 8 years of experience evaluated all angiographic images, with 20% randomly selected cases independently reviewed by a second radiologist to assess inter-rater reliability.

**Figure 1:**
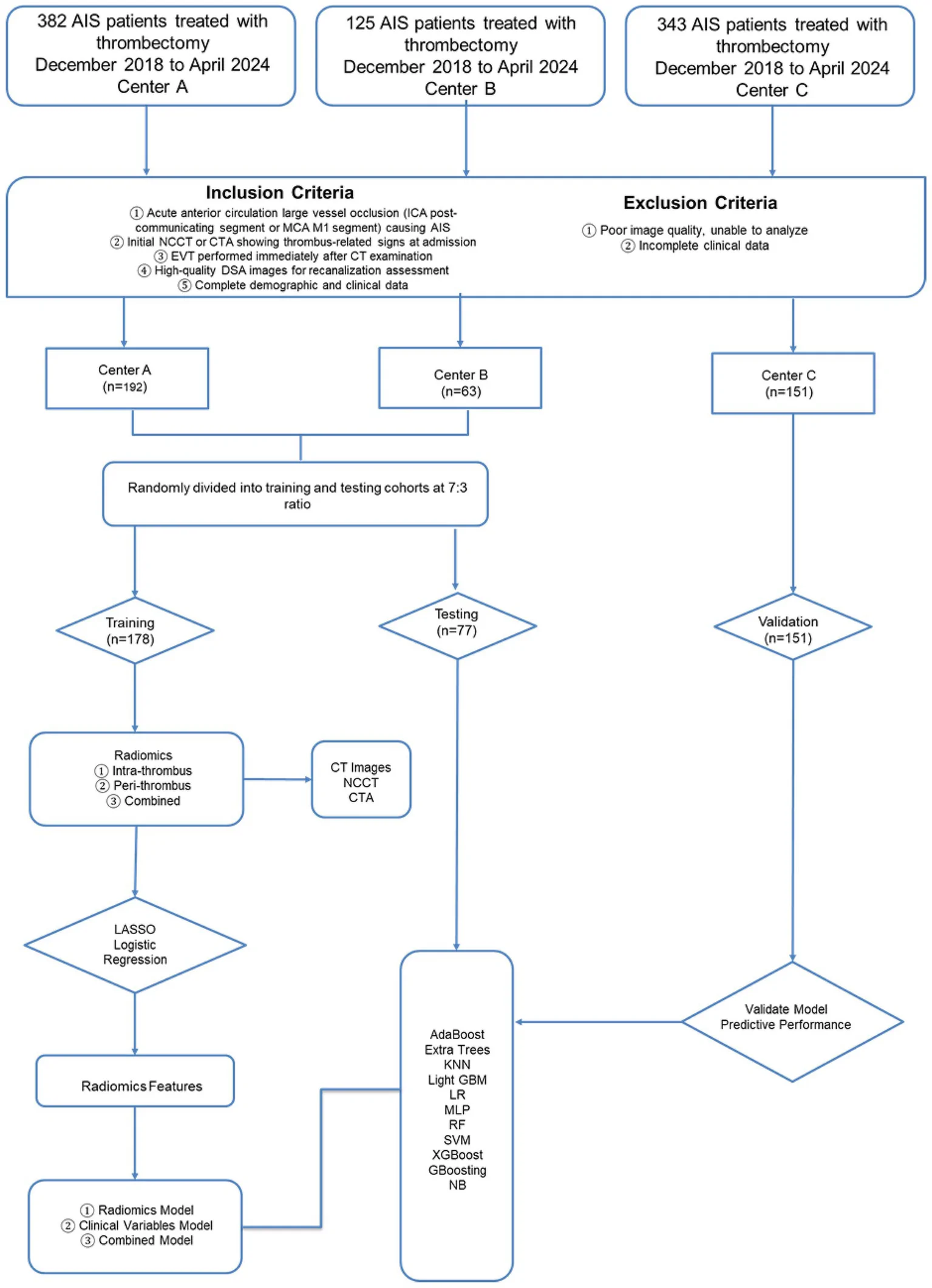
Flow chart of the patient-selection process.

Among 582 screened thrombectomy patients, 406 were enrolled after applying inclusion/exclusion criteria. Patients from Centers A and B were combined and randomly divided (7,3 ratio) into training (*n* = 178) and internal testing (*n* = 77) cohorts. Patients from Center C served as an independent external validation cohort (*n* = 151). The patient selection flowchart is shown in Figure 1.

### CT data acquisition and Thrombus segmentation

NCCT and CTA images were acquired using 64 to 256-slice CT scanners (SOMATOM Force, Siemens; Revolution CT/Optima CT 680, GE Healthcare), with reconstruction slice thickness of 0.63–1.00 mm. All images underwent intensity normalization (0–600 range) and isotropic resampling to 1x1x1 mm voxel resolution.

Using ITK-SNAP software (Version 3.6.0), thrombus delineation was performed referencing DSA images (19). CTA images were co-registered with DSA to localize proximal and distal thrombus boundaries. The thrombus watables manually delineated slice-by-slice on CTA and mapped to corresponding NCCT images. The perithrombus region was automatically generated by expanding the intrathrombus region of interest (ROI) outward by 1 mm using Python (Version 3.7.1). As we mentioned earlier (24, 25), the theoretical rationale for the 1-mm expansion of the perithrombus ROI is detailed below:(1) Anatomical basis: The medial layer of intracranial arteries normally measures 0.3–0.5 mm. A 1-mm outward margin fully encompasses the entire vessel wall and adjacent perivascular interstitial tissue, which is the core region where thrombus-vessel wall inflammation and vascular remodeling occur. Meanwhile, this narrow margin minimizes uneven contamination of brain parenchyma even between large-caliber ICA and relatively thinner MCA M1 segments.(2) Methodological consensus: Prior radiomics studies focusing on vascular lesions (carotid plaque, coronary artery CT imaging) widely adopt a 1–2 mm perilesional expansion as the standard setting.(3) Noise control: Expansion exceeding 2 mm will unavoidably incorporate remote brain parenchyma and cerebrospinal fluid, generating irrelevant imaging signals and distorting radiomic feature extraction. For reproducibility assessment, one radiologist randomly selected 30 thrombi for repeat delineation after 2 weeks, verified by a second radiologist. Both were blinded to clinical data.

### Feature extraction and selection

Radiomics features (*n* = 428) were extracted from intrathrombus and perithrombus ROIs on both NCCT and CTA using PyRadiomics library. Features encompassed first-order statistics, shape, and texture (GLCM, GLSZM, NGTDM, etc.). *Z*-score normalization was applied in the training cohort, with identical parameters applied to testing and validation cohorts.

Feature selection followed a three-step approach: (1) Mann–Whitney U test (*p* < 0.05) for univariate screening; (2) Spearman correlation (coefficient >0.9) for collinearity elimination (retaining features with smaller *p*-values); and (3) LASSO regression with 5-fold cross-validation to optimize the lambda parameter and identify the final feature subset (26, 27). For the combined model, features from both regions were pooled and subjected to identical selection procedures.

### Classifier model building and evaluation

**Figure 2:**
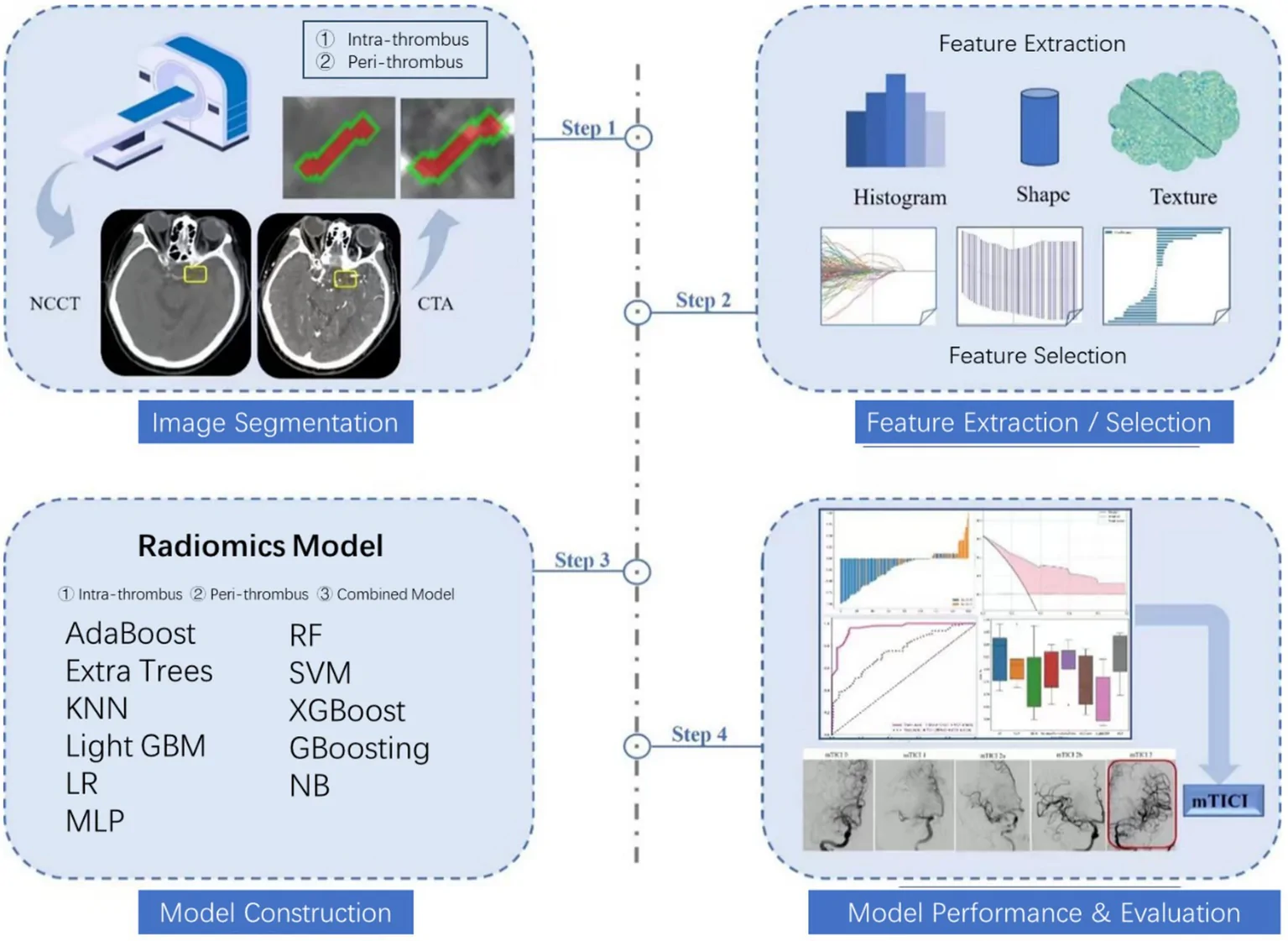
Study workflow of CT radiomics for predicting recanalization outcomes after endovascular thrombectomy.

Eleven supervised machine learning algorithms were evaluated: Adaptive Boosting (AdaBoost), Extra Trees, k-Nearest Neighbors (KNN), Light Gradient-Boosting Machine (LightGBM), Logistic Regression (LR), Multi-Layer Perceptron (MLP), Random Forest (RF), Support Vector Machine (SVM), eXtreme Gradient Boosting (XGBoost), Gradient Boosting (GBoosting), and Naive Bayes (NB). Models were developed for intrathrombus, perithrombus, and combined regions using 5-fold cross-validation. The study workflow is illustrated in Figure 2. This study adhered to the CheckList for EvaluAtion of Radiomics research (CLEAR) guidelines (28).

### Statistical analysis

Inter-rater reliability was assessed using intraclass correlation coefficient (ICC), with values >0.75 considered reliable (29). Model performance was evaluated using receiver operating characteristic (ROC) curve analysis, calculating AUC, sensitivity, specificity, positive predictive value (PPV), negative predictive value.

(NPV), and Matthews correlation coefficient (MCC) (30). The DeLong test compared ROC curves between models (31). Decision curve analysis assessed clinical utility (32). False discovery rate (FDR) correction was applied for multiple comparisons. Statistical significance was set at *p* < 0.05. Analyses were performed using SPSS 21.0 and Python 3.7.1.

## Results

### Patient clinical characteristics

Among 406 enrolled patients (mean age 67.4+/−12.3 years; 54.2% male), 219 (53.9%) achieved complete recanalization: 97 (54.5%) in training, 41 (53.2%) in testing, and 81 (53.6%) in validation cohorts. No significant differences were observed between complete and incomplete recanalization groups regarding age, sex, vascular risk factors, NIHSS score, thrombus location, or OPT across all cohorts (all *p* > 0.05), except for hyperlipidemia and coronary disease in the testing cohort (*p* = 0.02 and *p* = 0.03, respectively), which were adjusted for in subsequent analyses. Univariate and multivariate logistic regression revealed no statistically significant clinical predictors of complete recanalization.

The baseline clinical characteristics of patients across the three cohorts are summarized in Table 1. No significant differences were observed between complete and incomplete recanalization groups regarding age, sex, vascular risk factors, NIHSS score, thrombus location, or onset-to-puncture time across all cohorts.

**Table 1:** Clinical characteristics of patients.

Characteristic	Training (*n* = 178)	Testing (*n* = 77)	Validation (*n* = 151)	*P* (Train)	*P* (Test)	*P* (Val)
Age, years	70.8+/−12.1	65.5+/−12.8	70.8+/−11.6	0.06	0.44	0.39
Male, *n* (%)	104 (58.4)	52 (67.5)	84 (55.6)	0.58	0.17	0.61
Hypertension, *n* (%)	135 (75.8)	61 (79.2)	127 (84.1)	0.98	0.57	0.87
Hyperlipidemia, *n* (%)	28 (15.7)	11 (14.3)	22 (14.6)	0.92	0.02	0.75
Diabetes, *n* (%)	52 (29.2)	19 (24.7)	44 (29.1)	0.70	0.46	0.16
Smoking, *n* (%)	41 (23.0)	18 (23.4)	30 (19.9)	0.68	0.30	0.87
Atrial fibrillation, *n* (%)	52 (29.2)	26 (33.8)	44 (29.1)	0.70	0.42	0.75
Coronary disease, *n* (%)	36 (20.2)	18 (23.4)	33 (21.9)	0.96	0.03	0.27
NIHSS score	13.9+/−7.8	14.8+/−6.8	13.5+/−7.7	0.07	0.23	0.14
MCA location, *n* (%)	127 (71.3)	54 (70.1)	110 (72.8)	0.31	0.06	0.30
ICA location, *n* (%)	38 (21.3)	18 (23.4)	34 (22.5)	—	—	—
MCA + ICA, *n* (%)	13 (7.3)	5 (6.5)	7 (4.6)	—	—	—
OPT, min	320 (278–410)	302 (270–395)	338 (290–413)	0.58	0.11	0.74

Data are presented as mean +/− standard deviation or median (interquartile range). Categorical variables are expressed as number (percentage). ICA, internal carotid artery; MCA, middle cerebral artery; NIHSS, National institutes of health stroke scale; OPT, onset-to-puncture time. p values compare mTICI <=2b vs >2b groups within each cohort.

### Radiomics feature selection

**Figure 3:**
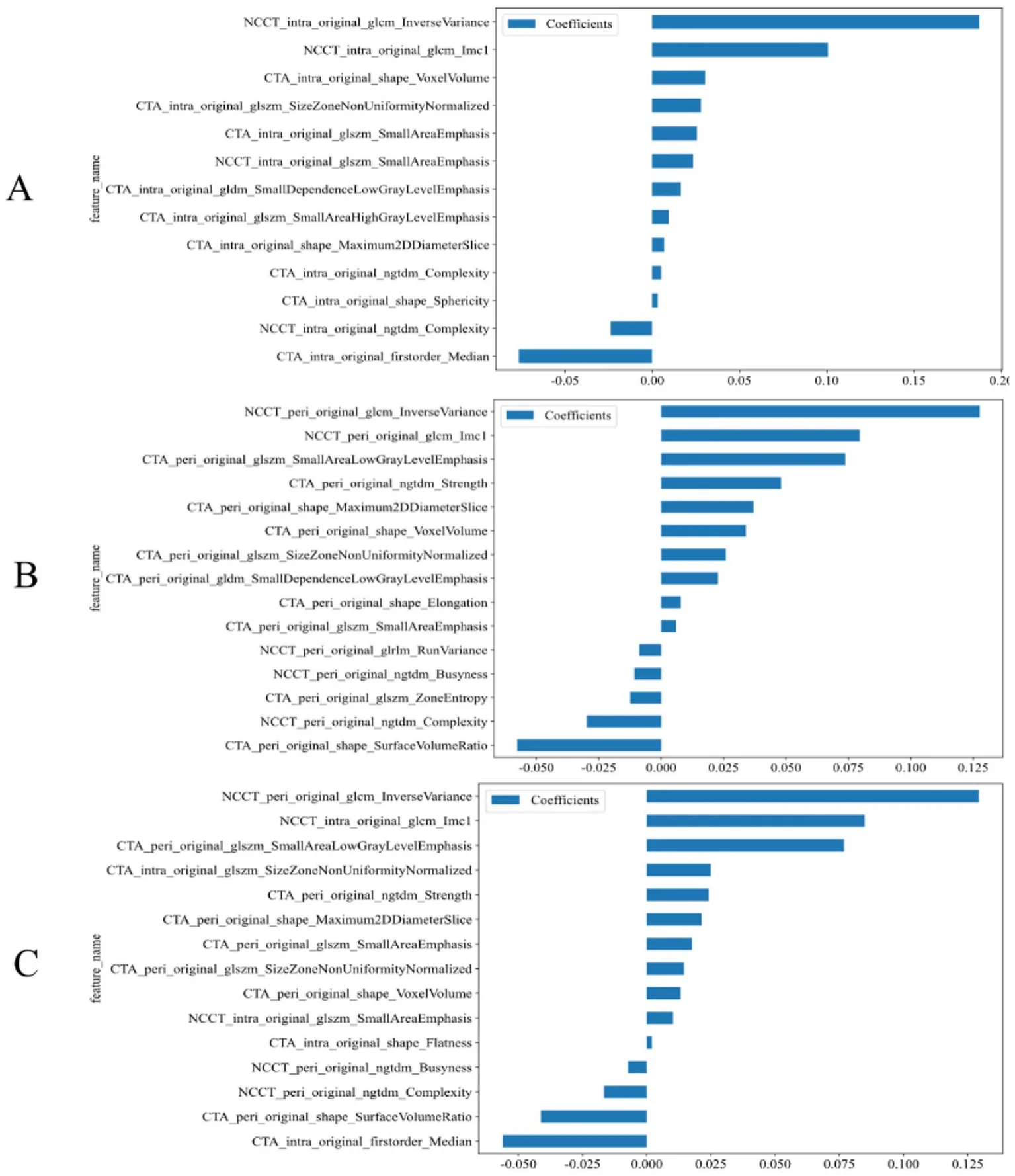
Histograms of radiomics scores (Rad-scores) for the selected features. **(A)** Distribution of the intrathrombus model (13 features), **(B)** perithrombus model (15 features), and **(C)** combined intrathrombus-perithrombus model (15 features). CC comparison across classifiers is presented in Figure 4.

Radiomics features demonstrated good inter-rater reliability (all ICC > 0.75). LASSO regression selected 13, 15, and 15 features for intrathrombus, perithrombus, and combined models, respectively. The combined model predominantly incorporated perithrombus features (10 of 15). Most selected features were derived from CTA (10 of 15) and belonged to shape and texture categories (GLSZM, NGTDM, GLCM). The radiomics score (Rad-score) was calculated as: Rad-score = 0.516854 + Sigma (Coefficient x Feature) (Supplementary Figure S1). The distribution of radiomics scores across the three models is shown in Figure 3 and Supplementary Table S3.

**Figure 4:**
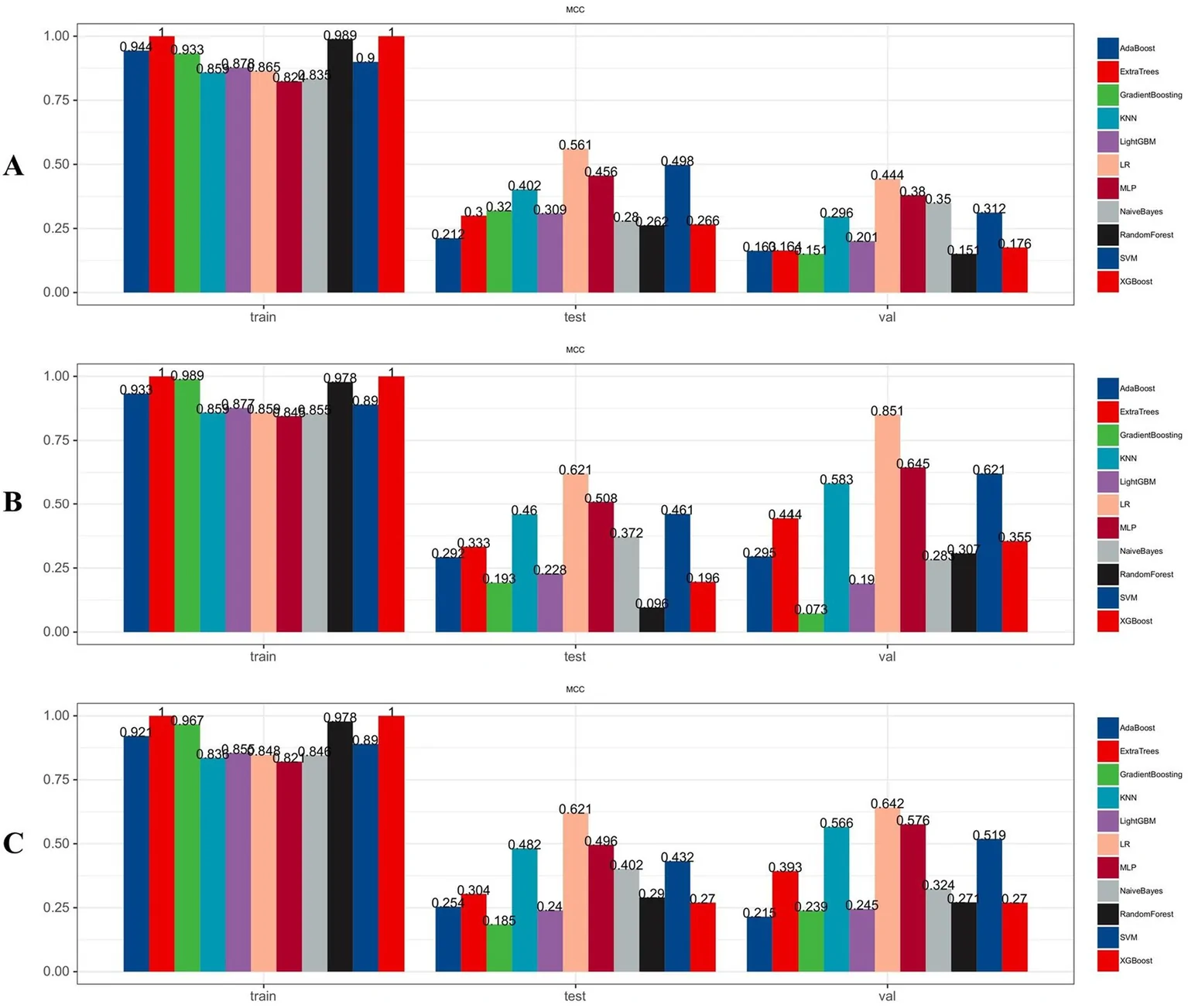
Bar plots of Matthews correlation coefficient (MCC) values for eleven classifiers across region-specific models and datasets. **(A)** Intra-thrombus model, **(B)** Peri-thrombus model, **(C)** Combined model.

### Model performance and comparison

In the training cohort, most classifiers achieved high AUC values (0.87–1.00). However, in testing and validation cohorts, complex models (AdaBoost, Extra Trees, XGBoost, and RF) exhibited substantial overfitting, with AUC values dropping to 0.52–0.79. In contrast, LR demonstrated stable performance across all cohorts: intrathrombus (AUC 0.73/0.77), perithrombus (AUC 0.82/0.85), and combined (AUC 0.88/0.86) for testing/validation cohorts, respectively. Performance comparison of different classifiers in Table 2 and Supplementary Table S3. *Of note,* Table 2 *only displays the full performance metrics of the intrathrombus LR model as a representative baseline case; complete LR performance data of the perithrombus and combined models across all cohorts are fully provided in* *Supplementary Table S3* *to simplify the main table layout.*

**Table 2:** Performance comparison of LR classifiers.

Classifier	Cohort	Accuracy	AUC	95% CI	Sensitivity	Specificity	PPV	NPV	F1	MCC
LR	Training	0.93	0.87	0.86–0.88	0.92	0.93	0.93	0.92	0.93	0.87
LR	Testing	0.77	0.73	0.70–0.76	0.78	0.76	0.74	0.80	0.76	0.56
LR	Validation	0.72	0.77	0.76–0.79	0.77	0.66	0.71	0.73	0.74	0.44

MCC values corroborated these findings. While Extra Trees and XGBoost achieved MCC 1.00 in training, they dropped to 0.16–0.44 in validation. LR achieved the highest MCC values in validation: 0.44 (intrathrombus), 0.85 (perithrombus), and 0.64 (combined). The M.

**Figure 5:**
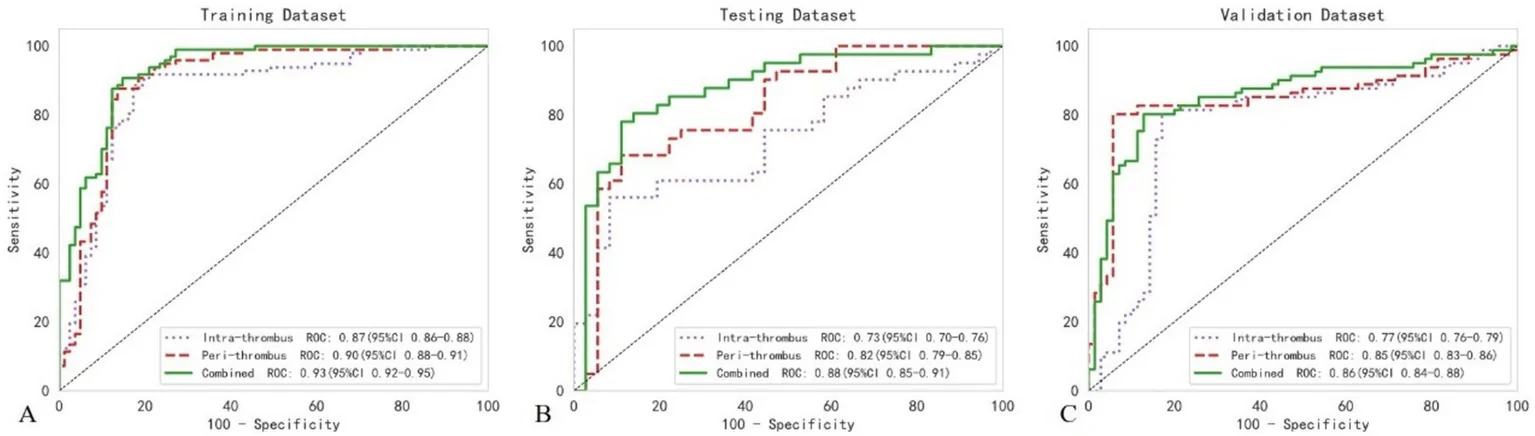
ROC curves of the logistic regression classifier in the training **(A)**, testing **(B)**, and validation **(C)** cohorts. The combined model (green) achieved AUCs of 0.93, 0.88, and 0.86, respectively. In the validation cohort, both perithrombus (0.85 vs. 0.77, *p* = 0.02) and combined models (0.86 vs. 0.77, *p* = 0.01) showed significantly higher accuracy than the intrathrombus model.

DeLong test comparisons revealed that in the training cohort, the combined model outperformed intrathrombus (AUC 0.93 vs. 0.87, *p* = 0.01) and perithrombus (AUC 0.93 vs. 0.90, *p* = 0.02) models. In testing, the combined model surpassed both intrathrombus (AUC 0.88 vs. 0.73, *p* < 0.001) and perithrombus (AUC 0.88 vs. 0.82, p = 0.02) models, with perithrombus superior to intrathrombus (*p* = 0.03). In validation, both perithrombus (AUC 0.85 vs. 0.77, p = 0.02) and combined (AUC 0.86 vs. 0.77, p = 0.01) models outperformed the intrathrombus model. The ROC curves for the logistic regression classifier are shown in Figure 5.

Decision curve analysis indicated that the combined model provided superior net benefit across threshold probabilities of 0.18–0.92 compared to treating all or none strategies.

## Discussion

This study developed and validated a CT radiomics model utilizing intrathrombus and perithrombus features to predict complete recanalization (mTICI 3) after EVT in AIS. The combined model with logistic regression achieved robust generalization (AUC 0.86 in external validation) and outperformed single-region models. Notably, perithrombus features dominated the combined model, highlighting the importance of vessel wall microenvironment characterization.

Previous work demonstrated that CT thrombus radiomics predicts successful recanalization (mTICI > = 2b) (20). However, mTICI > = 2b encompasses a broad spectrum of reperfusion quality (50–99%) with heterogeneous outcomes (21, 22). The present study specifically targets mTICI 2c/3, which is associated with significantly better 90-day functional outcomes than mTICI 2b (7, 21). By focusing on this stringent endpoint, our model addresses a more clinically relevant target that reflects the ideal angiographic goal of EVT.

The shift from predicting mTICI > = 2b to mTICI 2c/3 is methodologically significant. Residual stenosis or partial thrombus burden in mTICI 2b may obscure relationships between thrombus characteristics and recanalization mechanisms. Our model achieved AUC 0.86 for mTICI 2c/3 prediction despite this increased stringency, performance comparable to or exceeding prior models predicting the broader endpoint of successful recanalization (20, 33).

The superiority of the combined over intrathrombus-only model (AUC 0.86 vs. 0.77, *p* = 0.01) indicates that perithrombus features provide essential complementary information. This likely reflects the complex interaction between thrombus composition and vessel wall pathology. Histological studies demonstrate significant spatiotemporal heterogeneity in thrombus composition (platelets, fibrin, red blood cells), potentially reducing reliability of intrathrombus-only assessment (34, 35). Conversely, vessel wall changes are more stable and etiology-specific: atherosclerotic strokes show inflammatory infiltration and endothelial dysfunction, whereas cardioembolic strokes typically lack such changes (36, 37).

Complete recanalization depends not only on thrombus clearance efficacy but critically on underlying vessel wall pathology (38). Residual stenosis from atherosclerotic plaques or organized thrombus may prevent mTICI 2c/3 achievement despite partial thrombus removal. Perithrombus radiomics features capture vessel wall characteristics and thrombus-vessel interactions, enabling identification of patients with favorable conditions for complete flow restoration. This biological distinction explains why perithrombus features dominated our combined model (67% of selected features).

Evaluation of eleven classifiers revealed that LR outperformed complex algorithms (RF, XGBoost, and MLP) in generalization capability. While ensemble methods achieved high training accuracy (AUC 0.94–1.00), they exhibited substantial overfitting in external validation (AUC 0.52–0.79), likely due to sensitivity to data noise and high feature dimensionality relative to sample size (39).

LR’s linear nature reduces overfitting risk and enhances interpretability (40, 41). Given that radiomics features often exhibit linear relationships with outcomes in well-defined pathological contexts, parsimonious models like LR are preferable to black-box algorithms for thrombus characterization. This transparency facilitates clinical integration, allowing physicians to understand how specific texture and shape features contribute to predictions-critical for emergency stroke care decision-making.

The lack of improvement upon adding clinical variables suggests that radiomics features capture pathophysiological information beyond conventional assessment. Radiomics appears to sensitively detect subtle changes in thrombus permeability, heterogeneity, and vessel wall microenvironment invisible to visual inspection. The dominance of CTA-derived features (67%) likely reflects enhanced thrombus-vessel wall contrast from iodinated contrast agents, facilitating characterization of thrombus permeability and vessel wall integrity (42).

This study has limitations. First, the retrospective design may introduce selection bias; prospective validation is warranted. Second, manual ROI segmentation is time-consuming (5–10 min per case); future work should explore automated segmentation. Third, lack of histological analysis precludes direct correlation between radiomics features and thrombus biology. Fourth, we focused on mTICI 2c/3; future studies should explore differential prediction of mTICI 2b versus 3. Finally, long-term functional outcomes (modified Rankin Scale) were not available for this analysis.

## Conclusion

This study demonstrates that a combined CT radiomics model incorporating intrathrombus and perithrombus regions effectively predicts complete recanalization after EVT in AIS. The logistic regression classifier exhibited optimal generalization performance. These findings emphasize the importance of thrombus-vessel wall interactions in achieving complete recanalization and provide an objective tool for preoperative patient stratification and treatment optimization.

## Data Availability

The data analyzed in this study is subject to the following licenses/restrictions: the datasets are subject to restrictions due to patient privacy protection regulations and hospital data protection policies. The data were used under license from Shanghai Sixth People’s Hospital for the current study only. Access to the data is restricted and requires permission from the hospital. Requests to access these datasets should be directed to JJ, jjx2020@sjtu.edu.cn; HP, panine@sina.com; HT, tianhao1976@163.com.
