# Supplementary material for: CT-based intrathrombus and perithrombus radiomics for predicting complete recanalization after endovascular thrombectomy in acute ischemic stroke
Source: Front Neurol. 2026 Aug 19;17:1868821. doi: 10.3389/fneur.2026.1868821 (PMC13533805; doi:10.3389/fneur.2026.1868821)
Supplement: Supplementary file 1 [file Table_1.docx]

**Supplementary Information**

Radiomics (Rad) score was calculated as follows:

**Rad score = 0.516854 + Σ(coefficient × radiomics feature)**

**Supplementary Table 1. Baseline Clinical Characteristics of Patients**

| **Clinical Variable** | **TrainingCohort (n=178)** | **TrainingCohort (n=178)** | **TrainingCohort (n=178)** | **TestingCohort(n=77)** | **TestingCohort(n=77)** | **TestingCohort(n=77)** | **ValidationCohort(n=151)** | **ValidationCohort(n=151)** | **ValidationCohort(n=151)** |
| --- | --- | --- | --- | --- | --- | --- | --- | --- | --- |
| Clinical Variable | mTICI≤2b  (n=81) | mTICI＞2b  (n=97) | P value | mTICI≤2b  (n=36) | mTICI＞2b  (n=41) | P value | mTICI≤2b  (n=70) | mTICI＞2b  (n=81) | P value |
| Age，years | 68.95±12.54 | 72.42±11.30 | 0.06 | 64.22±12.62 | 66.46±12.91 | 0.44 | 69.86±11.65 | 71.49±11.56 | 0.39 |
| Sex,Male,n (%) | 45(55.56) | 59(60.82) | 0.58 | 21(58.33) | 31(75.61) | 0.17 | 41(58.57) | 43(53.09) | 0.61 |
| History, n(%) |  |  |  |  |  |  |  |  |  |
| Hypertension | 62(76.54) | 73(75.26) | 0.98 | 27(75.0) | 34(82.93) | 0.57 | 58(82.86) | 69(85.19) | 0.87 |
| Hyperlipidemia | 13(16.05) | 15(15.46) | 0.92 | 1(2.78) | 10(24.39) | 0.02 | 9(12.86) | 13(16.05) | 0.75 |
| Diabetes | 22(27.16) | 30(30.93) | 0.70 | 7(19.44) | 12(29.27) | 0.46 | 16(22.86) | 28(34.57) | 0.16 |
| Smoking | 17(20.99) | 24(24.74) | 0.68 | 6(16.67) | 12(29.27) | 0.30 | 14(20.0) | 16(19.75) | 0.87 |
| Atrial fibrillation | 22(27.16) | 30(30.93) | 0.70 | 10(27.78) | 16(39.02) | 0.42 | 19(27.14) | 25(30.86) | 0.75 |
| Coronary artery disease | 17(20.99) | 19(19.59) | 0.96 | 4(11.11) | 14(34.15) | 0.03 | 12(17.14) | 21(25.93) | 0.27 |
| NIHSS评分 | 12.60±6.87 | 15.03±8.43 | 0.07 | 13.75±6.77 | 15.63±6.82 | 0.23 | 14.56±8.24 | 12.69±7.28 | 0.14 |
| Thrombus location,n (%) |  |  | 0.31 |  |  | 0.06 |  |  | 0.30 |
| 1 MCA | 59(72.84) | 68(70.1) |  | 25(69.44) | 29(70.73) |  | 55(78.57) | 55(67.9) |  |
| 2 ICA | 14(17.28) | 24(24.74) |  | 11(30.56) | 7(17.07) |  | 12(17.14) | 22(27.16) |  |
| 3MCA+ ICA | 8(9.88) | 5(5.15) |  | 0(0.0) | 5(12.2) |  | 3(4.29) | 4(4.94) |  |
| OPT, min M(Q1-Q3) | 323(273-489) | 318 (281-358) | 0.58 | 317(275-445) | 287 (260-350) | 0.11 | 341 (296-425) | 334(283-403) | 0.74 |

**Supplementary Table 2. Radiomics Features Selected by LASSO Regression**

| **Model** | **Feature Category** | **Feature Category** | **Number of Features** | **Source** | **Source** |
| --- | --- | --- | --- | --- | --- |
| Model | Feature Category | Feature Category | Number of Features | CT | CTA |
| Intrathrombus | firstorder |  | 1 | 0 | 1 |
|  | shape |  | 3 | 0 | 3 |
|  | texture | GLCM | 2 | 1 | 1 |
|  | texture | GLSZM | 4 | 1 | 3 |
|  | texture | GLDM | 1 | 0 | 1 |
|  | texture | NGTDM | 2 | 1 | 1 |
| Perithrombus | firstorder |  | 0 | 0 | 0 |
|  | shape |  | 4 | 0 | 4 |
|  | texture | GLCM | 2 | 2 | 0 |
|  | texture | GLSZM | 4 | 0 | 4 |
|  | texture | GLDM | 1 | 0 | 1 |
|  | texture | NGTDM | 3 | 2 | 1 |
|  | texture | GLRLM | 1 | 1 | 0 |
| Combined | firstorder |  | 1(Peri-0 Intra-1) | 0 | 1 |
| （Intrathrombus+Perithrombus） | shape |  | 4(Peri-3 Intra-1) | 0 | 4 |
|  | texture | GLCM | 2(Peri-1 Intra-1) | 2 | 0 |
|  | texture | GLSZM | 5(Peri-3 Intra-2) | 1 | 4 |
|  | texture | NGTDM | 3(Peri-3 Intra-0) | 2 | 1 |

**Supplementary Table 3. Performance Comparison of Different Classifiers**

| **Classifier** | **Cohort** | **Accuracy** | **AUC** | **95% CI** | **Sensitivity** | **Specificity** | **PPV** | **NPV** | **F1** | **MCC** |
| --- | --- | --- | --- | --- | --- | --- | --- | --- | --- | --- |
| Intrathrombus |  |  |  |  |  |  |  |  |  |  |
| AdaBoost | Training | 0.97 | 0.99 | 0.99 - 1.00 | 0.98 | 0.95 | 0.96 | 0.98 | 0.97 | 0.94 |
| AdaBoost | Testing | 0.60 | 0.59 | 0.46 - 0.72 | 0.39 | 0.78 | 0.61 | 0.59 | 0.48 | 0.21 |
| AdaBoost | Validation | 0.58 | 0.52 | 0.43 - 0.62 | 0.71 | 0.44 | 0.57 | 0.58 | 0.63 | 0.16 |
| ExtraTrees | Training | 0.48 | 1.00 | 1.00 - 1.00 | 0.00 | 1.00 | 0.00 | 0.48 | NaN | 1.00 |
| ExtraTrees | Testing | 0.56 | 0.66 | 0.54 - 0.78 | 0.39 | 0.71 | 0.54 | 0.57 | 0.45 | 0.30 |
| ExtraTrees | Validation | 0.56 | 0.57 | 0.48 - 0.67 | 0.74 | 0.37 | 0.56 | 0.57 | 0.64 | 0.16 |
| GBoosting | Training | 0.96 | 0.99 | 0.99 - 1.00 | 0.96 | 0.97 | 0.97 | 0.95 | 0.96 | 0.93 |
| GBoosting | Testing | 0.65 | 0.66 | 0.54 - 0.78 | 0.58 | 0.71 | 0.64 | 0.66 | 0.61 | 0.32 |
| GBoosting | Validation | 0.56 | 0.55 | 0.45 - 0.64 | 0.35 | 0.78 | 0.63 | 0.53 | 0.45 | 0.15 |
| KNN | Training | 0.89 | 0.98 | 0.97 - 0.99 | 0.80 | 0.98 | 0.97 | 0.82 | 0.88 | 0.86 |
| KNN | Testing | 0.65 | 0.74 | 0.63 - 0.85 | 0.39 | 0.88 | 0.74 | 0.62 | 0.51 | 0.40 |
| KNN | Validation | 0.60 | 0.66 | 0.57 - 0.74 | 0.49 | 0.71 | 0.64 | 0.57 | 0.56 | 0.30 |
| LightGBM | Training | 0.93 | 0.98 | 0.97 - 1.00 | 0.89 | 0.98 | 0.98 | 0.89 | 0.93 | 0.88 |
| LightGBM | Testing | 0.64 | 0.61 | 0.48 - 0.74 | 0.36 | 0.88 | 0.72 | 0.61 | 0.48 | 0.31 |
| LightGBM | Validation | 0.58 | 0.53 | 0.44 - 0.62 | 0.87 | 0.27 | 0.56 | 0.67 | 0.68 | 0.20 |
| LR | Training | 0.93 | 0.87 | 0.86 - 0.88 | 0.92 | 0.93 | 0.93 | 0.92 | 0.93 | 0.87 |
| LR | Testing | 0.77 | 0.73 | 0.70 - 0.76 | 0.78 | 0.76 | 0.74 | 0.80 | 0.76 | 0.56 |
| LR | Validation | 0.72 | 0.77 | 0.76 - 0.79 | 0.77 | 0.66 | 0.71 | 0.73 | 0.74 | 0.44 |
| MLP | Training | 0.90 | 0.93 | 0.89 - 0.98 | 0.86 | 0.95 | 0.95 | 0.86 | 0.90 | 0.82 |
| MLP | Testing | 0.71 | 0.78 | 0.67 - 0.88 | 0.72 | 0.71 | 0.68 | 0.74 | 0.70 | 0.46 |
| MLP | Validation | 0.67 | 0.68 | 0.59 - 0.76 | 0.89 | 0.44 | 0.63 | 0.78 | 0.73 | 0.38 |
| NB | Training | 0.90 | 0.94 | 0.90 - 0.98 | 0.82 | 1.00 | 1.00 | 0.84 | 0.90 | 0.84 |
| NB | Testing | 0.62 | 0.63 | 0.50 - 0.75 | 0.67 | 0.59 | 0.59 | 0.67 | 0.62 | 0.28 |
| NB | Validation | 0.67 | 0.67 | 0.59 - 0.76 | 0.71 | 0.63 | 0.67 | 0.67 | 0.69 | 0.35 |
| RF | Training | 0.98 | 1.00 | 1.00 - 1.00 | 0.97 | 1.00 | 1.00 | 0.97 | 0.98 | 0.99 |
| RF | Testing | 0.58 | 0.59 | 0.46 - 0.72 | 0.14 | 0.98 | 0.83 | 0.56 | 0.24 | 0.26 |
| RF | Validation | 0.54 | 0.56 | 0.47 - 0.65 | 0.47 | 0.62 | 0.57 | 0.52 | 0.52 | 0.15 |
| SVM | Training | 0.94 | 0.99 | 0.97 - 1.00 | 0.91 | 0.98 | 0.98 | 0.91 | 0.94 | 0.90 |
| SVM | Testing | 0.70 | 0.73 | 0.61 - 0.84 | 0.92 | 0.51 | 0.62 | 0.88 | 0.74 | 0.50 |
| SVM | Validation | 0.64 | 0.63 | 0.54 - 0.72 | 0.87 | 0.38 | 0.60 | 0.74 | 0.71 | 0.31 |
| XGBoost | Training | 0.99 | 1.00 | 1.00 - 1.00 | 0.99 | 1.00 | 1.00 | 0.99 | 1.00 | 1.00 |
| XGBoost | Testing | 0.62 | 0.63 | 0.50 - 0.75 | 0.47 | 0.76 | 0.63 | 0.62 | 0.54 | 0.27 |
| XGBoost | Validation | 0.55 | 0.52 | 0.42 - 0.61 | 0.94 | 0.14 | 0.54 | 0.67 | 0.68 | 0.18 |
| Perithrombus |  |  |  |  |  |  |  |  |  |  |
| AdaBoost | Training | 0.96 | 0.99 | 0.99 - 1.00 | 0.95 | 0.98 | 0.98 | 0.94 | 0.96 | 0.93 |
| AdaBoost | Testing | 0.64 | 0.65 | 0.53 - 0.78 | 0.47 | 0.78 | 0.65 | 0.63 | 0.55 | 0.29 |
| AdaBoost | Validation | 0.59 | 0.66 | 0.58 - 0.75 | 0.35 | 0.85 | 0.71 | 0.55 | 0.47 | 0.30 |
| ExtraTrees | Training | 0.48 | 1.00 | 1.00 - 1.00 | 0.00 | 1.00 | 0.00 | 0.48 | NaN | 1.00 |
| ExtraTrees | Testing | 0.62 | 0.68 | 0.57 - 0.80 | 0.78 | 0.49 | 0.57 | 0.71 | 0.66 | 0.33 |
| ExtraTrees | Validation | 0.70 | 0.78 | 0.71 - 0.85 | 0.64 | 0.77 | 0.75 | 0.67 | 0.69 | 0.44 |
| GBoosting | Training | 0.99 | 1.00 | 0.99 - 1.00 | 0.99 | 0.99 | 0.99 | 0.99 | 0.99 | 0.99 |
| GBoosting | Testing | 0.58 | 0.53 | 0.40 - 0.66 | 0.56 | 0.61 | 0.56 | 0.61 | 0.56 | 0.19 |
| GBoosting | Validation | 0.51 | 0.50 | 0.40 - 0.59 | 0.33 | 0.70 | 0.54 | 0.50 | 0.41 | 0.07 |
| KNN | Training | 0.92 | 0.98 | 0.96 - 0.99 | 0.86 | 0.99 | 0.99 | 0.87 | 0.92 | 0.86 |
| KNN | Testing | 0.69 | 0.76 | 0.65 - 0.86 | 0.72 | 0.66 | 0.65 | 0.73 | 0.68 | 0.46 |
| KNN | Validation | 0.77 | 0.84 | 0.78 - 0.91 | 0.74 | 0.80 | 0.80 | 0.74 | 0.77 | 0.58 |
| LightGBM | Training | 0.93 | 0.98 | 0.97 - 1.00 | 0.95 | 0.92 | 0.93 | 0.94 | 0.94 | 0.88 |
| LightGBM | Testing | 0.60 | 0.62 | 0.50 - 0.75 | 0.64 | 0.56 | 0.56 | 0.64 | 0.60 | 0.23 |
| LightGBM | Validation | 0.59 | 0.61 | 0.52 - 0.70 | 0.63 | 0.55 | 0.60 | 0.58 | 0.61 | 0.19 |
| LR | Training | 0.92 | 0.90 | 0.88 - 0.91 | 0.87 | 0.98 | 0.98 | 0.88 | 0.92 | 0.86 |
| LR | Testing | 0.78 | 0.82 | 0.79 - 0.85 | 0.92 | 0.66 | 0.70 | 0.90 | 0.80 | 0.62 |
| LR | Validation | 0.91 | 0.85 | 0.83 - 0.86 | 0.99 | 0.84 | 0.87 | 0.98 | 0.92 | 0.85 |
| MLP | Training | 0.92 | 0.94 | 0.90 - 0.98 | 0.88 | 0.95 | 0.95 | 0.88 | 0.92 | 0.85 |
| MLP | Testing | 0.74 | 0.79 | 0.68 - 0.89 | 0.75 | 0.73 | 0.71 | 0.77 | 0.73 | 0.51 |
| MLP | Validation | 0.82 | 0.85 | 0.81 - 0.93 | 0.87 | 0.75 | 0.79 | 0.85 | 0.83 | 0.65 |
| NB | Training | 0.92 | 0.95 | 0.91 - 0.98 | 0.89 | 0.95 | 0.95 | 0.89 | 0.92 | 0.86 |
| NB | Testing | 0.68 | 0.73 | 0.61 - 0.84 | 0.56 | 0.78 | 0.69 | 0.67 | 0.62 | 0.37 |
| NB | Validation | 0.62 | 0.66 | 0.57 - 0.74 | 0.41 | 0.84 | 0.73 | 0.57 | 0.53 | 0.28 |
| RF | Training | 0.98 | 1.00 | 1.00 - 1.00 | 0.96 | 1.00 | 1.00 | 0.96 | 0.98 | 0.98 |
| RF | Testing | 0.52 | 0.52 | 0.39 - 0.65 | 0.36 | 0.66 | 0.48 | 0.54 | 0.41 | 0.10 |
| RF | Validation | 0.60 | 0.68 | 0.60 - 0.76 | 0.30 | 0.92 | 0.79 | 0.55 | 0.43 | 0.31 |
| SVM | Training | 0.94 | 0.97 | 0.94 - 1.00 | 0.90 | 0.98 | 0.98 | 0.90 | 0.94 | 0.89 |
| SVM | Testing | 0.71 | 0.77 | 0.66 - 0.87 | 0.75 | 0.68 | 0.68 | 0.76 | 0.71 | 0.46 |
| SVM | Validation | 0.80 | 0.84 | 0.80 - 0.92 | 0.74 | 0.86 | 0.85 | 0.76 | 0.80 | 0.62 |
| XGBoost | Training | 0.99 | 1.00 | 1.00 - 1.00 | 0.99 | 1.00 | 1.00 | 0.99 | 1.00 | 1.00 |
| XGBoost | Testing | 0.58 | 0.59 | 0.46 - 0.72 | 0.58 | 0.59 | 0.55 | 0.62 | 0.57 | 0.20 |
| XGBoost | Validation | 0.66 | 0.68 | 0.60 - 0.77 | 0.89 | 0.41 | 0.62 | 0.77 | 0.73 | 0.36 |
| Combined |  |  |  |  |  |  |  |  |  |  |
| AdaBoost | Training | 0.94 | 0.99 | 0.98 - 1.00 | 0.92 | 0.95 | 0.96 | 0.92 | 0.94 | 0.92 |
| AdaBoost | Testing | 0.61 | 0.60 | 0.47 - 0.73 | 0.31 | 0.88 | 0.69 | 0.59 | 0.42 | 0.25 |
| AdaBoost | Validation | 0.59 | 0.61 | 0.52 - 0.70 | 0.42 | 0.77 | 0.66 | 0.55 | 0.52 | 0.22 |
| ExtraTrees | Training | 0.48 | 1.00 | 1.00 - 1.00 | 0.00 | 1.00 | 0.00 | 0.48 | NaN | 1.00 |
| ExtraTrees | Testing | 0.66 | 0.71 | 0.59 - 0.82 | 0.47 | 0.83 | 0.71 | 0.64 | 0.57 | 0.30 |
| ExtraTrees | Validation | 0.68 | 0.77 | 0.70 - 0.84 | 0.55 | 0.81 | 0.75 | 0.63 | 0.64 | 0.39 |
| GBoosting | Training | 0.98 | 1.00 | 0.99 - 1.00 | 0.96 | 1.00 | 1.00 | 0.96 | 0.98 | 0.97 |
| GBoosting | Testing | 0.58 | 0.59 | 0.45 - 0.72 | 0.67 | 0.51 | 0.55 | 0.64 | 0.60 | 0.19 |
| GBoosting | Validation | 0.61 | 0.63 | 0.55 - 0.72 | 0.80 | 0.41 | 0.59 | 0.65 | 0.68 | 0.24 |
| KNN | Training | 0.89 | 0.97 | 0.95 - 0.99 | 0.80 | 0.99 | 0.99 | 0.83 | 0.89 | 0.84 |
| KNN | Testing | 0.64 | 0.75 | 0.64 - 0.86 | 0.31 | 0.93 | 0.79 | 0.60 | 0.44 | 0.48 |
| KNN | Validation | 0.72 | 0.81 | 0.74 - 0.88 | 0.56 | 0.88 | 0.83 | 0.65 | 0.67 | 0.57 |
| LightGBM | Training | 0.92 | 0.98 | 0.97 - 1.00 | 0.88 | 0.95 | 0.95 | 0.88 | 0.92 | 0.86 |
| LightGBM | Testing | 0.61 | 0.63 | 0.51 - 0.76 | 0.53 | 0.68 | 0.59 | 0.62 | 0.56 | 0.24 |
| LightGBM | Validation | 0.62 | 0.63 | 0.55 - 0.72 | 0.60 | 0.63 | 0.64 | 0.60 | 0.62 | 0.25 |
| LR | Training | 0.92 | 0.93 | 0.92 - 0.95 | 0.86 | 0.98 | 0.98 | 0.87 | 0.91 | 0.85 |
| LR | Testing | 0.78 | 0.88 | 0.85 - 0.91 | 0.92 | 0.66 | 0.70 | 0.90 | 0.80 | 0.62 |
| LR | Validation | 0.82 | 0.86 | 0.84 - 0.88 | 0.83 | 0.80 | 0.81 | 0.82 | 0.82 | 0.64 |
| MLP | Training | 0.90 | 0.95 | 0.92 - 0.98 | 0.88 | 0.93 | 0.93 | 0.88 | 0.91 | 0.82 |
| MLP | Testing | 0.73 | 0.77 | 0.66 - 0.88 | 0.81 | 0.66 | 0.67 | 0.79 | 0.73 | 0.50 |
| MLP | Validation | 0.78 | 0.83 | 0.77 - 0.90 | 0.80 | 0.77 | 0.79 | 0.78 | 0.79 | 0.58 |
| NB | Training | 0.92 | 0.95 | 0.92 - 0.98 | 0.87 | 0.97 | 0.96 | 0.87 | 0.91 | 0.85 |
| NB | Testing | 0.69 | 0.72 | 0.61 - 0.84 | 0.53 | 0.83 | 0.73 | 0.67 | 0.61 | 0.40 |
| NB | Validation | 0.62 | 0.67 | 0.58 - 0.75 | 0.36 | 0.90 | 0.80 | 0.57 | 0.50 | 0.32 |
| RF | Training | 0.97 | 1.00 | 1.00 - 1.00 | 0.94 | 1.00 | 1.00 | 0.94 | 0.97 | 0.98 |
| RF | Testing | 0.56 | 0.63 | 0.51 - 0.76 | 0.14 | 0.93 | 0.63 | 0.55 | 0.23 | 0.29 |
| RF | Validation | 0.62 | 0.68 | 0.60 - 0.76 | 0.36 | 0.89 | 0.78 | 0.57 | 0.49 | 0.27 |
| SVM | Training | 0.94 | 0.97 | 0.95 - 1.00 | 0.90 | 0.98 | 0.98 | 0.90 | 0.94 | 0.89 |
| SVM | Testing | 0.70 | 0.74 | 0.63 - 0.85 | 0.72 | 0.68 | 0.67 | 0.74 | 0.69 | 0.43 |
| SVM | Validation | 0.74 | 0.81 | 0.75 - 0.88 | 0.62 | 0.88 | 0.84 | 0.68 | 0.71 | 0.52 |
| XGBoost | Training | 0.99 | 1.00 | 1.00 - 1.00 | 0.99 | 1.00 | 1.00 | 0.99 | 1.00 | 1.00 |
| XGBoost | Testing | 0.62 | 0.65 | 0.53 - 0.78 | 0.58 | 0.66 | 0.60 | 0.64 | 0.59 | 0.27 |
| XGBoost | Validation | 0.63 | 0.66 | 0.57 - 0.75 | 0.64 | 0.62 | 0.64 | 0.62 | 0.64 | 0.27 |

*Note: AUC, area under the curve; CI, confidence interval; PPV, positive predictive value; NPV, negative predictive value; MCC, Matthews correlation coefficient; LR, logistic regression; LASSO, least absolute shrinkage and selection operator; NCCT, non-contrast CT; CTA, CT angiography; ICA, internal carotid artery; MCA, middle cerebral artery; NIHSS, National Institutes of Health Stroke Scale; mTICI, modified Thrombolysis in Cerebral Infarction; OPT, onset-to-puncture time.*
